# Comment on “Utilizing %Carbohydrate-Deficient Transferrin as a Biomarker to Complement Interviews in Stratifying Alcohol Consumption in Patients with Alcohol Dependence: Aiming for Application to Fatty Liver Disease”

**DOI:** 10.31662/jmaj.2025-0398

**Published:** 2025-11-21

**Authors:** Amnuay Kleebayoon, Viroj Wiwanitkit

**Affiliations:** 1 Private Academic Consultant, Samraong, Cambodia; 2 Department of Research Analytics, Saveetha Dental College and Hospitals, Saveetha Institute of Medical and Technical Sciences Saveetha University, Chennai, India

**Keywords:** transferrin, biomarker, alcohol, dependence

## Dear Editor,

The publication on “Utilizing %carbohydrate-deficient transferrin as a biomarker to complement interviews in stratifying alcohol consumption in patients with alcohol dependence: aiming for application to fatty liver disease ^(1)^” is hereby discussed. This cross-sectional study included 285 serum samples from patients treated at two specialized alcohol dependence clinics. While the sample size was adequate for biomarker assessment, pre-selection of patients with a diagnosis of alcohol dependence may have resulted in biases in the distribution of alcohol consumption levels, limiting its applicability to the general population, such as social drinkers or patients with indiscriminate consumption. Furthermore, while %carbohydrate-deficient transferrin (%CDT) is a widely established marker, its sensitivity varies depending on genetics, metabolism, and gender, all of which were not accounted for or thoroughly investigated in this investigation. Furthermore, the statistical study, which relied exclusively on p-values and did not include confidence intervals, effect sizes, or receiver operating characteristic (ROC) studies, lacked clinical validity for the recommended cutoff values (1.67% and 2.48%).

Despite the limitations of the research methodology, the findings point to a dose-dependent connection. There is a dose-dependent relationship between alcohol consumption and %CDT. However, the inability of gamma-glutamyl transferase (GGT) and the GGT-CDT index to discriminate consumption below 30 g/day for men and 20 g/day for women highlights the limitations of screening for moderate drinking, which can have long-term health repercussions. A%CDT of 1.67% could be a useful threshold for discriminating between low-risk and moderate-risk patients, allowing for early treatment planning. A 2.48% threshold corresponds to heavy alcohol intake, which increases the CDT’s reliability as a binary screening tool. Another intriguing possibility is that %CDT could be used as a dynamic biomarker to track drinking behavior or treatment response, rather than a static diagnostic criterion.

Based on the evidence presented here, the following questions should be addressed for future study: How do age, gender, ethnicity, and liver comorbidities affect %CDT values? Should cutoff levels be modified based on population groups? Furthermore, in comparison to emerging biomarkers like phosphatidylethanol, which have higher sensitivity and specificity than %CDT at low to moderate consumption levels, can %CDT still play a clinical role? Is it possible to add %CDT into the new quantitative criteria for the classification of fatty liver disease (steatotic liver disease [SLD]), hence minimizing reliance on manual history taking? Finally, may artificial intelligence or machine learning technologies be used to develop predictive models that include %CDT, GGT, and other biochemical indicators to improve drinking level assessment?

To improve the therapeutic usefulness of %CDT, future study should construct a prospective cohort with a diverse population and a control group free of alcohol dependence to accurately determine baseline values and variability in CDT. Follow-up over time will enable the examination of CDT changes based on drinking behavior, such as abstinence or relapse. Furthermore, ROC analysis can be utilized to find the optimal cutoff value in a clinical setting. Furthermore, models that combine CDT with other biochemical indicators, psychosocial assessments, and imaging technologies, such as liver stiffness measurement, should be created. (Elastography) may facilitate more complete treatment of alcoholic liver disease (SLD).

## Article Information

### Author Contributions

Amnuay Kleebayoon 50 % ideas, writing, analyzing, approval. Viroj Wiwanitkit 50 % ideas, supervision, approval

### Conflicts of Interest

None

### Data Availability Statement

There is no new data generated.

### AI Declaration

Computational tool is use for language editing in preparation of the manuscript.

## References

[1] Iwasa M, Eguchi A, Suzuki T, et al. Utilizing %carbohydrate-deficient transferrin as a biomarker to complement interviews in stratifying alcohol consumption in patients with alcohol dependence: aiming for application to fatty liver disease. JMA J. 2025;8(3). 10.31662/jmaj.2025-0109 PMC12328905 40786492

